# Parents’ experiences of accessing respite care for children with Autism Spectrum Disorder (ASD) at the acute and primary care interface: a systematic review

**DOI:** 10.1186/s12887-020-02045-5

**Published:** 2020-05-22

**Authors:** Emma Cooke, Valerie Smith, Maria Brenner

**Affiliations:** Trinity College Dublin, Dublin, Ireland

**Keywords:** Autism Spectrum disorder, Autism, Autistic disorder, Respite care, Unscheduled care, Short break, Children, Accessing respite care, Integrated care

## Abstract

**Background:**

Population prevalence estimates by the World Health Organisation suggest that 1 in 160 children worldwide has an Autism Spectrum Disorder (ASD). Accessing respite care services for children with an ASD can often be a daunting and exhaustive process, with parents sometimes forced to access acute hospital services as an initial point of contact for respite care or in a crisis situation. To gain an in-depth understanding of accessing respite care for children with an ASD, from the perspectives of parents, a systematic review of the evidence on parent’s experiences and views of respite care for children with an ASD at the acute and primary interface was undertaken.

**Methods:**

Pubmed, Embase, CINAHL and PsycINFO were systematically searched. Studies identified as relevant based on predetermined eligibility criteria were selected for inclusion. The search strategy also targeted unpublished studies and grey literature. Qualitative data and qualitative components of mixed method studies that represented the experiences of parents accessing respite care for children with an ASD were eligible for inclusion. A meta-aggregative approach was used during data synthesis.

**Results:**

Database searching elicited 430 records of which 291 studies remained after removal of duplicates. These 291 studies were screened for title and abstract by two reviewers resulting in 31 studies to be screened at full text and assessed for eligibility. Six studies met the inclusion criteria and a further additional study also met the inclusion criteria during a manual search. As a result, 7 studies were selected for the review as set out in Fig. 1.

**Conclusion:**

In the absence of appropriate services and defined pathways to support services such as respite care, overwhelmed parents and community providers of mental health resources may not be in a position to meet the specific needs of children with an ASD and their families which may be contributing to a direct increase in hospitalizations. This systematic review identified a number of barriers to respite care, of which the findings can be used to inform future service development and further research. Knowledge of parental experiences in caring for a child with an ASD is vital in addressing the need and type of respite care required for children with an ASD.

**Systematic review registration:**

PROSPERO CRD42018106629.

## Background

The provision of care closer to home for children with neurodevelopmental needs is being increasingly recognised both nationally and internationally. This is particularly the case for children with and an Autism Spectrum Disorder (ASD). ASD is a lifelong neurological condition characterized by unusual behaviors and impairments in communication skills and social interactions [1]. Population prevalence estimates by the World Health Organisation suggest that 1 in 160 children worldwide has an Autism Spectrum Disorder (ASD) [2]. Parents are, in most cases, the primary caregivers for these children and caring for a child with an ASD can be a full-time job. Consequently, there is a heightened economic, social and political need to address the issue of integrated care and provision of support services such as respite care for a growing population of children with ASD.

The upbringing of a child with an ASD is complex and associated with an important change in family dynamics [3]. There is considerable evidence that raising a child with an ASD can be a source of increased family stress [4–7]. Interestingly, mothers of children with an ASD experience chronic stress comparable to that experienced by combat soldiers [8]. In addition to typical parenting demands, parents of children with an ASD also have extra demands related to caring for their child’s often unpredictable behaviour and emotional challenges. Parents can take on complex care tasks such as behaviour and aggression management while balancing all other aspects of family life and work commitments. The adverse effect of this reality is burn-out within a short period of time [9]. Therefore, the complexity of the parental role and well-being of parents caring for children with an ASD is increasingly becoming a public health issue [10–12].

Respite care can be defined as the provision of care to children with complex care needs for a specific period of time with the intent of providing temporary relief to the main carers and their family [13]. Often, despite initial reluctance to use respite services, parents find respite care beneficial [14]. Research reflects the many benefits of respite services for parents such as significant improvement in psychological adjustment, fatigue, mental health and quality of life [15]. In a survey of 122 single mothers of children with autism, 59.8% of mothers accessed respite care and 71% reported that they were satisfied with this care [16]. However, the majority of the respite care was provided informally by family members and friends which could have influenced the high satisfaction rate. Similarly, 88.6% of parents of children with autism reported being satisfied with their care providers which primarily comprised grandparents, babysitters, community agencies and extended family members [17]. Accessing respite care can often be challenging as a result of complex systems to access services and lack of knowledge about existing services available. There can often be confusion over points of accessing care and no defined system of documenting care needs and care delivery in a manner that can be accessible for the family and the multi-disciplinary team across acute and primary care services [18]. Consequently, parents may be forced to access acute hospital services as an initial point of contact or in a crisis situation [19–21]. One study found that children with an ASD were nine times more likely to visit an emergency department compared with children who do not have an ASD as a result of psychiatric problems such as physical aggression, disruptive behaviour, running away and hurting oneself [22]. Another study including children with and without an ASD classified about a third of the paediatric emergency room visits by children with ASD as “inappropriate” because the problems could have been handled in an outpatient setting [23]. As a result, parents accessing acute hospital care services as an initial point of access for services such as respite care can be considered a symptom of inadequate service provision for children with an ASD.

There is limited evidence of the actual experience of families seeking access to respite care services for children with an ASD. Furthermore, little is known about the profile and numbers of children with an ASD accessing respite care. Motivating factors for parents attending acute hospitals as a means of seeking respite care for children is multi-factorial and needs to be explored in order to identify and address the current gaps in primary care service provision. In order to improve integrated care services at the acute and primary care interface it is necessary to explore what is currently happening by examining the literature, exploring the pathways to access respite care and exploring the experiences and perspectives of parents accessing respite care for their child with ASD.

This systematic review question is centred around asking what is the available evidence on access to respite care from the perspectives of parents of children with an ASD and what are the key issues, if any, that parents may encounter when accessing respite care for their child.

## Methods

A systematic review protocol was developed using the Joanna Briggs Institute Method for Systematic Review Research [24]. The PRISMA checklist was used to guide the reporting of the review [25]. The review protocol was registered on the PROSPERO International prospective register of systematic reviews. (Registration number CRD42018106629).

### Eligibility criteria

Eligibility criteria included parents of children with an ASD who had accessed respite care and offered their views and experiences of the process through qualitative enquiry. The focus on children was influenced by the potential of increasing needs for children with ASDs from childhood through to adolescence which can make them and their parents susceptible to physical and mental health crises because their specific needs are often unmet [26]. There is considerable evidence that the upbringing of a child with an ASD is complex and associated with an important change in family dynamics and family stress is higher in families raising a child with an ASD [4–7]. In addition to typical parenting demands, parents of autistic children also have extra demands related to caring for their child’s often unpredictable behaviour and emotional challenges. Consequently, this can lead to a greater need for accessing respite care while the child is growing up and its is likely that parents will start seeking the need for respite as the child begins to get older.

Rationale for focusing on qualitative studies included the need to gain a holistic view of participant lived experiences and the phenomenon under study [27] while also figuring out how meanings are shaped through and in culture [28]. As participants lived experience cannot always be counted and measured through the use of quantitative methods, qualitative studies offered better access to ‘how’ and ‘why’ a particular phenomenon, or behaviour, operates as it does in a particular context. Furthermore, qualitative methodology is beneficial where there is limited knowledge on a phenomenon which is applicable in the context of the research topic. Studies that focused on qualitative data including, but not limited to, designs such as phenomenology and grounded theory, were included. Mixed method studies were also included with relevant data related to the qualitative component of the study only, extracted. The age of the child had to be below 18 years. Unpublished studies and ‘grey literature’ of relevance to the review question were also considered.

### Search strategy

A broad search strategy was applied to maximise the potential for capturing all relevant data and to minimize the potential for non-retrieval of relevant studies. The following keywords and search terms derived broadly from a combination of the review’s population and exposure inclusion criteria were used; Autism Spectrum Disorder, Autism, Autistic Disorder, Respite care, unscheduled care, short break, Children, Accessing Respite Care, Integrated Care. Databases searched included Cumulative Index to Nursing and Allied Health Literature (CINAHL+) (via EBSCOhost), PsycINFO (via EBSCOhost), PubMed and Embase, with the same search strategy used in each database. All databases were searched from their date of inception to the date of the search. However, no limitation was placed on the age of the data. The first known paper on autism was published by Kanner in 1943 [29]. To meet the aims of a broad exploratory systematic review, studies published in English, during any publication period were accepted. A manual search of the reference list of all articles included at full text level was also conducted to identify potential studies that may not have been captured by searching the databases. The search strategy also targeted unpublished studies and ‘grey literature’ to help minimize the risk of missing unpublished studies. Numerical search results were reported using the PRISMA flow diagram [25]. Covidence was used to record citations, abstracts and full texts and to identify duplicates. The search was first conducted in May 2018 and repeated again in August 2018. The search was repeated again in December 2019 and found no new studies matching the inclusion criteria.

### Study selection

Study selection was performed using a two-stage process; i) screening of title and abstracts only, excluding studies ineligible at this stage based on predefined inclusion criteria, and forwarding those that appeared relevant or where uncertainty existed around their relevance, for review of their full-texts; ii) retrieval and assessment of full-texts for inclusion in the review. Two reviewers independently screened titles and abstracts of all citations retrieved during the search, after removal of duplicate citations, for eligibility. Disagreements that arouse between the reviewers were resolved through discussion and consensus.

### Appraisal of study quality

All included studies were formally assessed for methodological rigor, using a critical appraisal tool from Brunton et al., [30], by the author and a second independent reviewer assessed approximately 50% of the selected studies. Issues considered during the appraisal process included rigor, credibility and relevance of studies selected to the systematic review question. As part of the quality appraisal process, studies were summarized in table form, showing the study and reference, aim/objectives, sampling and recruitment, characteristics of participants, socio-economic status, validity and reliability of data collection methods, tools and relevance to the systematic review. The overall quality of each included study was reported as high, moderate or low based on the results of the critical appraisal process.

## Results

**Fig. 1:**
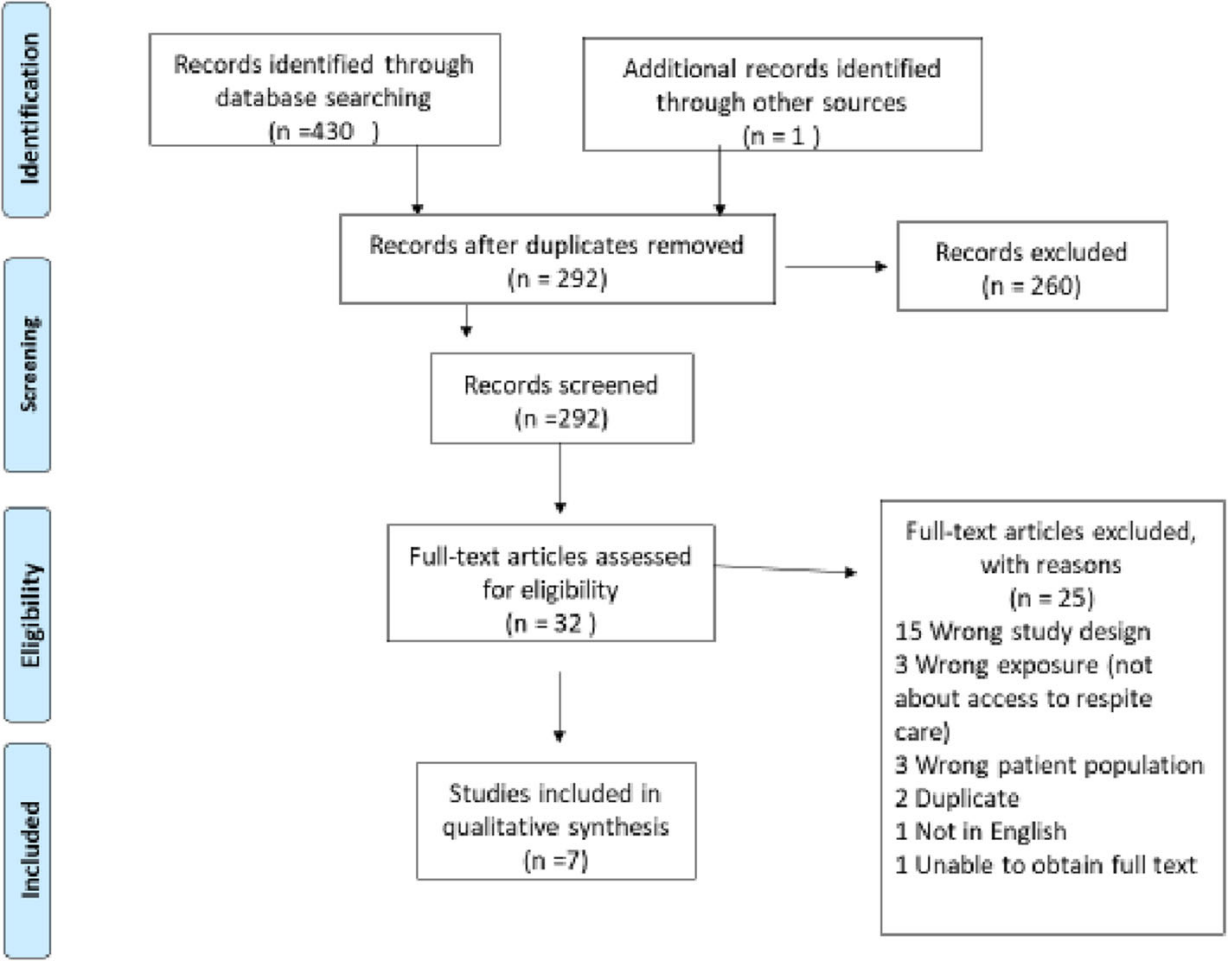
Flow chart of search outcomes and included studies (PRISMA) [25]

Database searching elicited 430 records of which 291 studies remained after removal of duplicates (139). These 291 studies were screened for title and abstract by two reviewers resulting in 31 studies. Of these 31 studies, 25 were excluded after full text review for the following reasons; wrong study design (15), wrong exposure (3), wrong patient population (3), duplication (2), not in English (1), unable to obtain full text (1). Communication was made with the author of the one study that could not be obtained, however, there was no response. This resulted in six studies meeting the inclusion criteria and were included in the final review. At this stage the reference lists of identified papers were searched manually to identify possible omitted studies. One additional study that met the inclusion criteria was sourced during the manual search. As a result, 7 studies were selected for the review as set out in Fig. 1. Interestingly, no single study was found that exclusively explored the views of parents of children with ASD accessing respite care, rather this aspect was a component within the studies.

Seven articles were appraised in total. Six studies were reported as high quality and one study was reported as medium. No studies were found to be of low quality. The author and independent reviewer reached a 97% agreement on the independent quality appraisal assessment of a 50% sample of included studies.

### Participants and settings

One study was conducted in Pakistan and India. Two studies were conducted in Ireland and the remaining studies were conducted in Sweden, Kenya, Canada, and the United States. The profile of participants included a mixture of mothers, parents, families, couples. Six of the studies employed a qualitative design and one study employed a mixed methods design of which the qualitative datum was extracted only for the purpose of this review. The profile of participants from the included studies is detailed in Table 1. The majority of children with ASD were male. Some of the children had been newly diagnosed with autism [31] and other children, in addition to their autism were described as having either a learning disability or an intellectual disability characterized by limitations in intellectual functioning (e.g learning, reasoning, problem solving skills) and/or adaptive behaviour (conceptual, social and practical skills) which was classified as moderate to profound within some studies. Table 1 outlines the profile of participants from the chosen studies.

**Table 1:** Profile of participants from included studies

Author and Year	Number of Parents	Profile of Parents mother/father	Number of Children	Gender of child	Age of Children	Disability	Characteristics
Benderix et al., 2007	5 couples	Not specified	5	Not specified	10-11 years	Learning disability moderate ×1 severe ×3 bprofound × 1	Delayed verbal/absent language, lacked spontaneous seeking of enjoyment, little or no interest in other persons, hyperactivity, short attention span, self-injurious behaviour, high pain threshold, disorders of sleep.
Gona et al., 2016	103 parents (& HCP’s)	Not specified	Not specified	Not specified	Not specified	Not specified	Not specified
Hartrey 2003	1	mother	1	female	8 years	intellectual regression	Rett Syndrome, scoliosis, anti-social, constipation, reflux, teeth grinding
Hodgetts et al. 2013	9	mothers (8) father (1)	7 children (under the age of 19) (2 aged 19&29)	Male	6-10 years	All had moderate to severe intellectual disability.	3 had some functional language, 2 did not have functional language. 4 individuals were diagnosed with Asperger syndrome, had mild or no intellectual impairment, and demonstrated conversational language skills.
Mann 2013	13	13 mothers	13	Male (11) Female (2)	4-17 yrs	Disability not specified. Autism described as Moderate (4), mild (3), severe and ADHD (2) Mild to mod (1)	*as reported by mothers
Minhas et al. 2015	15	mothers (11) fathers (4)	Not specified	Male (12) Female (3)	Not specified	Not specified	Not specified
Keenan 2007	95 parents and carers	87 (female = 4 grandmothers and 3 mothers) 8 (male)	100	Male (80) Female (20)	mean age 8 years 30 (1-6 yrs) 56 (7-12 yrs) 11 (13-15 yrs) 3 (16 yrs)	Intellectual disability: 56 with learning disability ranging from mild, moderate and severe	ASD (90) Aspergers (7) PDD (2) Unknown (1)

### Data extraction and synthesis

Details from eligible studies were extracted using a pre-designed data extraction spreadsheet detailing study characteristics such as publication year, study design, country of origin, methodology, sample size, data collection and data analysis procedures, point of access to respite care, type of respite care, conclusion, key findings. This also allowed for synthesis of methodologies and methods. A meta-aggregative approach was used during data synthesis. The meta-aggregative method is aligned with the philosophy of Hannes and Lockwood [32] where the meaning is connected to the idea of practical usefulness and pragmatism and supports the pragmatic approach to this systematic review. Furthermore, the mega-aggregative method has been developed in order to deliver usable synthesized findings to inform decision making at the clinical or policy level. Therefore, this approach was considered helpful when attempting to explore current practice on accessing respite care from the perspectives of parents of children with autism.

The results synthesized during meta-aggregation were the qualitative findings related to parents of children with an ASD and views/experiences of access to respite care. The aim of meta-synthesis by meta-aggregation was to assemble findings from qualitative research, categorize those findings into groups on the basis of similarity in meaning and aggregate these to generate a set of statements that adequately represent that aggregation. Meta-aggregation was achieved using NVivo software through the following three step approach;

1. Extraction of all findings from all included studies related to views/experiences of access to respite care only.
2. Developing categories for findings with at least two findings per category.
3. Developing one or more synthesized findings from at least two categories.

Categorization involved repeated, detailed examination of the extracted findings. The author identified groups of findings on the basis of similarity in meaning to create categories. A category is the combination of a brief description of a key concept arising from the aggregation of two or more similar findings. This description is accompanied by an explanatory statement that conveys the whole inclusive meaning of a group of similar findings.

The results are presented based on the synthesised findings that emerged. Validation of data extracted, and themes identified from all studies was carried out by two reviewers together. Synthesis of the included studies data identified three prominent themes that reflected the perspectives of parents of children with an ASD on accessing respite care. These include (1) The journey to getting and maintaining respite care, (2) The role of healthcare professionals in supporting parents to access services including respite care, and (3) Facilitating factors and barriers to accessing respite services.

### The journey to accessing and maintaining respite care

Parents’ experience of accessing respite care was described as a relentless journey. A total of four stages emerged from the synthesis as part of the process of accessing and maintaining respite care services for children with an ASD. Although these stages were not necessarily sequential, each stage featured within the overall journey to accessing respite care. The stages included; identifying the need for/motivating factors for seeking respite care, accessing and maintaining respite care services, parental concerns about respite care services, and alternative options to respite care.

One of the motivating factors for seeking respite was associated with an inability to manage behavioural problems which directly caused increased stress levels amongst parents [31, 33]. Parents reported that as the child grew stronger, they became more difficult to manage [34]. Parents began to seek help outside the home when the secondary behavioural problems associated with an ASD became visible and disrupted family life. ‘We thought he would grow out of it, but he has become worse. Now if he gets upset he bites himself, bangs his head on the wall and if somebody goes near him he gets even more upset. No one comes near him’ (father of an 8-year-old boy, urban Rawalpindi) [35].

One study found that the strongest predictor of stress for parents was associated with the existence of aggression in a child with ASD [36]. Consequently, high levels of stress emerged as the greatest motivator for accessing respite care [31, 33–35, 37]. However, finding and accessing respite care was equally identified as a stressor [36]. Findings identified a relationship between parents’ ability to have respite care services and their stress levels. For example, in one study, mothers frequently described high levels of stress because of the lack of any respite. ^‘^I can’t think of a time when I enjoyed a social occasion ... she is getting worse, and it has started to affect my health’ (mother of a 5-yearold girl, urban Rawalpindi) [35]. ‘How can I look after her when I have so much to do, sometimes I just leave her on her own ... then I feel guilty .. I don’t know what to do’ (mother of a 6-year-old girl, rural Rawalpindi) [35].

A number of studies outlined how family members were the main source of respite care for parents [34, 35, 37]. In the absence of any external support, parents described how spouses, siblings, grandparents and extended family members provided the main respite for the mothers and often families moved closer together so that the extended family could provide the support [33, 35]. Two studies reported that state-provided respite care was non-existent [31, 35]. Those availing of respite care services were receiving some respite care via supported housing [36] residential/group home/supported housing, [37] and boarding school [37]. One study described the various types of respite services available to participants including out-of-home respite, in-home respite, home-to-home scheme, holiday club and after school club [37]. Other families were in receipt of direct payments to choose from different respite care options for their child [33]. In the absence of any formalised support system or state funded respite care, traditional healers were citied in one study as being the first port of call for both rural and urban parents in the context of being a source of guidance and relief for carers [35]. These were identified as religious, traditional healers or herbal practitioners and others involved in magic or sorcery.

Accessing respite care services in rural areas was described as a challenge for parents [31, 35]. The most commonly cited reason was that services were located mainly in major cities which were at a distance and parents could not afford the financial or time costs [31, 35]. Overall, parents outlined a significant requirement for respite care but could not access this. Some parents described how they had attempted for many years through the government to obtain adequate home based respite care services for their children in order to be able to manage the family situation but had not succeeded and resorted to arranging a specialised group home in a building that the government made available [34]. Parents also described alternative methods used to find respite care such as online advertisements, newspapers, posting at local universities, grocery stores and word a mouth.

For parents that had accessed and were receiving respite care, the outcome or the impact was described in terms of both the child getting respite and the family [38].

Some parents felt that respite services placed too much emphasis on the medical model and valued a more social care model delivered by healthcare professionals [34]. For example, parents reported wanting a stronger educational orientation and less nursing and for the children to be given more training [34] as parents hoped their child would become more independent and receive lifelong school training or a meaningful daily occupation. Parents also valued non-pharmacological behaviour management strategies recommended by healthcare professionals such as psychologists, behaviourists and occupational therapists [36].

The benefits of respite care were described and reported in all studies. However, parents in one study described how they were not satisfied with their form of respite care, which was provided through a group home, as they felt that their child did not get enough of the activities they needed. The group home was described as a specialized group home made available by the municipality for children with autism and learning disabilities in which the parents could leave their child for respite care ranging from weeks to days [34].

Equally parents acknowledged that no longer having control over their child caused stress and anxiety [34]. The child refusing to return to respite care also caused stress and anxiety and would prevent the parent from sending their child back [34]. Despite experiencing positive outcomes as a result of availing of respite, parents still expressed ambivalent feels towards leaving their child. Strong attachments to their children rooted in their child’s dependency made availing of respite care and the associated short term removal of their child difficult and led to a sense of guilt [34].

### The role of healthcare professionals in seeking respite care services and additional supports

With the exception of one [38], all studies mentioned the role of healthcare professionals in the context of seeking support services including respite care or the impact of healthcare professionals on the child and family while in receipt of respite care services [31, 33–37]. Parents in one study described how they were made to feel inferior by healthcare professionals when deciding to seek home and family respite support for their children and families [33]. Healthcare professionals’ refusing access to respite care was also cited by some parents as their child did not meet predefined criteria [33]. Families also reported that professionals they met did not appreciate what they went through on a daily basis [33]. Almost half the parents in one study had not been informed by statutory services about home and respite supports available to their family [33]. Parents felt not knowing the questions to ask healthcare professionals prevented them from accessing the appropriate support services [33].

Parents reported that the medical profession had only limited information or knowledge about ASD and a lack of understanding about the condition [35, 36] which often impacted on their ability to provide appropriate supports such as respite care. Parents spoke about how doctors sometimes focused on providing medication as a primary source of intervention [35] and did not consider alternative options. Parents in receipt of respite services for their child valued continuity with staff [35] but also reported that a lack of experienced staff in respite centres was a concern to them [36].

### Facilitating factors and barriers to accessing respite care services for children with autism

Perceived barriers to respite care were described at a systems level such as lack of information to assist parents and a lack of general support services for children with an ASD [31, 33, 37]. The uncertainty of the autism condition and lack of adequate information from medical professionals was also described as a barrier to accessing respite care.

Parents wanted a shift from the medical model to a more social model of care within respite care services [34]. Seeing their child pleased was reassuring for parents but knowing that their child is being taken care of properly enabled parents to have confidence in the respite care service. Noticing an improvement in their child indicated that their child was happy in respite care which had a positive impact on the parents [34].

## Discussion

Children with an ASD primarily live with their families and research has shown that parenting a child with an ASD can produce stress in the family. All the studies in the systematic review described the stress associated with caring for a child with an ASD. Stress caused by the child’s aggression and an inability to cope with challenging behaviour was found to be one of the greatest motivating factors for parents seeking respite care.

Respite care is an important support service for parents of children with an ASD. In the current review, there was a reported lack of respite care for children with an ASD. It is clear that the sociocultural, demographic, economic and health systems context is quite different from high income countries, where most interventions for such conditions are researched and developed. Parents and families have demonstrated great resilience when faced with challenges accessing respite care services for their children with an ASD. Overall, this review found that families were the biggest providers of respite care for parents of children with an ASD. This reflects research findings throughout the literature.

The point at which parents began to seek respite care services for a child with an ASD was an important finding in this review, with the need for respite care services often increasing as children with an ASD enter adolescence and coincides with increased physical size and strength. A critical need for both behavioural and crises intervention has also been identified. Better access to behavioural health services designed for children with an ASD, such as specialized care coordination and respite care is needed to reduce the burden on acute services and prevent parents from seeing acute services as their only source of meaningful help and access to support services. This is supported by Mandell et al. who examined the use of respite care of over 28,000 children and young adults (through age 21) in the United States with an autism diagnosis and found that based on procedure costs, each $1000 increase in spending on respite care during the preceding 60 days resulted in an 8% decrease in the odds of hospitalization [39]. Liu et al. examined the profile on emergency department utilization in adolescents with autism and found that over 20% of children were accessing emergency services as a result of behavioural problems [40]. Overall, further research of service utilization before and after emergency department visits for children with autism is needed to develop a more effective intervention for children and parents in need of access to support services such as respite care.

An immense need for, but an inability to access respite care was a prominent finding of this systematic review. Similarly, the limitations of respite care reported in the literature are primarily associated with accessing respite care and can be attributed to the level of information about the availability of respite services [41], inflexibility in services, a lack of choice of services [42] and geographical location [43]. Pickard and Ingersoll also identify similar barriers to accessing respite care, such as finances, access to information, transportation and waiting lists [44]. Identified principles in best practice for the provision of respite suggest that there be a single point of access to respite care services in a given administrative area. National and international reports share a common need which centres around a range of appropriate options and supports to meet the diversity of need amongst people with an ASD and to ensure that service provision is responsive, accessible, integrated, co-ordinated, seamless and delivered in partnership with service users and families [45]. However, availability and range of services can often depend on the priority given to respite care by health and social service authorities and these limitations are often a consequence of a lack of resources.

Disparities with accessing respite services in rural areas was also a finding of this review which is shared nationally and internationally. Dew et al. reported that caregivers of individuals with autism living outside of metropolitan centres in Australia noted a lack of autism expertise in healthcare providers in rural and remote areas of the country [41]. Similarly, Thomas et al. (2012) found that caregivers of children with autism who lived in rural communities in the United States reported significantly less access to special summer camps and respite care [46]. In Ireland, the Slainte Healthcare Report [47] outlined that there remained significant geographic differences in access to respite care services in the general sense. Furthermore, the report suggests that based on the evidence, there are often long wait times to access rationed services without choice of service provider and that they also end up paying out of pocket for such services [47]. The financial burden of caring for a child with an ASD was also reported by parents in five out of the seven included studies in this systematic review [31, 33, 35–37]. Parents in receipt of direct payments to access respite support services or respite care grants found this approach very beneficial [33].

Research suggests that respite care services need to be accessible, affordable, in a location that is convenient, provided at the right time, in the right duration and in the right frequency [21, 48–50]. One study referenced how their child’s multidisciplinary home-based supports were discontinued because of their son’s aggression [36]. This finding is significant as while parents in general throughout the selected studies found that respite care was hard to access, parents in this particular study found it more difficult to maintain because of their child’s aggression. This was attributed to both a lack of appropriate care givers and a perceived lack of adequate funding by the government. Therefore, respite care services also need to be responsive and adaptive to changes in a child’s behaviour with an ASD. Having the capacity and capability to meet a family and child’s changing needs should also be a criterion for delivering effective respite care services by enabling the family and child to maintain services rather than remove services.

The benefits of respite care were described and reported throughout the studies in this review. Research supports the need for respite care to be more than just the physical separation of a caregiver from their child [21, 50]. The findings from this review suggest that the quality of respite care should be equally as important as the quantity of respite care and this should be considered when improving access to respite care services for children with an ASD. Furthermore, respite programs should be evaluated based on quality and satisfaction and subjected to an accreditation process to ensure providers of respite care are adequately trained to provide for the complex needs of children with an ASD.

The diagnosis and treatment of ASD occurs in multiple settings and is provided by variety of health professionals including family physicians, pediatricians, neurologists, psychiatrists, psychologists and speech & language therapists. As a result, healthcare professionals play a fundamental role in supporting parents to access support services including respite care for their child with an ASD. A significant finding that emerged from the synthesis of data were parental feelings that professionals either lacked knowledge surrounding ASDs or had limited understanding as to what resources they should refer for parents and caregivers. A survey of autism knowledge and attitudes among healthcare professionals found that current professionals in the field have an unbalanced understanding of autism due to presence of several misconceptions regarding many of the common features of autism including developmental, cognitive and emotional features [51]. The study found that more than a quarter of study participants were not likely to endorse the need for vital support and intervention services. This can have significant implications as a lack of awareness of services required for children with an ASD may also suggest that healthcare professionals could be less likely to advocate for much needed services for children with ASDs such as respite care services.

A study found that caregivers of children and adolescents with autism report they are more likely to access acute services for healthcare when they perceived their healthcare providers do not listen to their concerns, display cultural insensitivity, do not supply needed information, and do not involve caregivers in decision making [40]. This reflects the findings of this review which identified how some parents were made to feel inferior by healthcare professionals when deciding to seek home and family respite support for their children and families. In addition, healthcare professionals refusing access to respite care was also identified. Consequently, parents of children with an ASD may be less inclined to access their primary care physicians when seeking access to respite care as a result of having less assurance for help with acute, emergent, or complex behavioural or health issues [52]. These findings emphasize the importance of professionals to be connected and up to date with resources, services, and trainings to help parents feel better supported.

### Implications for health services

Service providers and funding agencies need to better plan and provide respite services to families with children who have an ASD. Healthcare professional education could include the development and provision of continuing education courses aimed at increasing the awareness of the problems associated with parenting a child with an ASD. Respite care services should centre around a range of appropriate options and supports to meet the diversity of need amongst children with an ASD. Furthermore, respite care services for children with autism should be responsive, accessible, integrated, co-ordinated, seamless and delivered in partnership with service users and families. Access to information on respite care services for children with an ASD needs to be transparent and made available to parents and caregivers throughout their engagements with healthcare professionals.

### Research implications

Further research is needed concerning implied outcomes and cost of supplying respite care to parents of children with an ASD. There appears to be little research on effective models of respite care for children with an ASD. In addition, further exploration on the benefits of respite care on outcomes for individuals is warranted. The findings from this research could also be enhanced by more quantitative or mixed methods research aimed at investigating the child perspectives or evaluating the impact of interventions such as increasing respite care staff training in challenging behaviour or building workforce capacity. Further research is also needed to examine the effectiveness of respite care services and the economic benefits of an accessible, flexible and integrated system of services.

### Strengths and limitations of the review

This review identified and included seven published studies only, of which none exclusively explored the issue of access to respite care, thus the data informing the meta-synthesis and the overall review’s findings, while narratively rich, were limited. Respite care has been identified as a challenge for systematic reviewers, particularly regarding how best to identify appropriate evidence for inclusion [53]. The variety of need, type of breaks and range of impacts leads to difficulties when attempting to evaluate outcomes and effectiveness of services provided. A lack of thorough evaluation around the benefits of respite care has been attributed to concerns around existing research methodologies, ethical issues, sample sizes and the use of inappropriate outcome measures. A significant strength of this review, however, is that included studies were assessed as methodologically robust (high quality), which adds weight to, and increases confidence in the validity and reliability of the findings for informing care, practice and policy. However, it must be noted that not all children with an ASD require the same needs as respite care. While a number of studies did indicate the severity level of children with an ASD, others did not. Therefore, not all the results obtained in the reviewed studies are necessarily comparable to all families of children with an ASD.

A possible limitation to the methodology of this review is that inter-reliability checks were not carried out after validation of extracted data and identified themes.

## Conclusion

This systematic review synthesized the available evidence on access to respite care from the perspectives of parents of children with an ASD and reported on the quality of the findings.

Respite care was viewed by parents as a vital resource to assist in the management of their child with an ASD, however, access to respite services is a significant challenge, and parents are prepared to go to extreme lengths to obtain appropriate respite services for their children. Findings highlighted that the provision of respite care may not be solely sufficient to alleviate care giver burden. One way of alleviating this is to consider the impact and benefit of providing respite within the home to reduce some of the distress experienced by parents and potential separation guilt often associated with respite care services. However, such a particular continuity of delivery would pose a challenge to services and warrants further consideration.

In the absence of appropriate services and defined pathways to support services such as respite care, overwhelmed parents and community providers of mental health resources may not be in a position to meet the specific needs of children with an ASD and families which may be contributing to a direct increase in hospitalizations. This review discovered several barriers to respite care, all of which could be addressed in future service provision and research. Knowledge of parental experiences in caring for a child with autism is vital in addressing the need and type of respite care required for children with an ASD.

## Data Availability

The datasets used and/or analysed during the current study are available from the corresponding author on reasonable request.

## Abbreviations

CINAHL: Cumulative Index of Nursing and Allied Health Literature
PRISMA: Preferred Reporting Items for Systematic Reviews and Meta-Analyses
ASD: Autism Spectrum Disorder SCIE
WHO: World Health Organization

## References

[1] American Psychiatric Association (2013). Diagnostic and statistical manual of mental disorders (DSM– 5).

[2] WHO (2017). World Health Organisation. Autism Spectrum Disorders.

[3] Cridland EK, Jones SC, Magee CA, Caputi P (2014). Family-focused autism spectrum disorder research: A review of the utility of family systems approaches. Autism..

[4] Hayes SA, Watson SL (2013). The impact of parenting stress: A meta-analysis of studies comparing the experience of parenting stress in parents of children with and without autism spectrum disorder. J Autism Dev Disord.

[5] Silva L, Schalock M (2012). Autism parenting stress index: Initial psychometric evidence. J Autism Dev Disord.

[6] Siman-Tov A, Kaniel S (2011). Stress and personal resource as predictors of the adjustment of parents to autistic children: A multivariate model. J Autism Dev Disord.

[7] Zablotsky B, Bradshaw C, Stuart E (2013). The association between mental, stress and coping supports in mothers of children with autistic spectrum disorder. J Autism Dev Disord.

[8] Smith LE, Hong J, Seltzer MM, Greenberg JS, Almeida DM, Bishop S (2010). Daily experiences among mothers of adolescents and adults with autism spectrum disorder. J Autism Dev Disord.

[9] Woodgate RL, Edwards M, Ripat JD, Borton B, Rempe G (2015). Intense parenting: a qualitative study detailing the experiences of parenting children with complex care needs. BMC Pediatr.

[10] Smith J, Swallow V, Coyne I (2015). Involving parents in managing their child’s long-term condition-A concept synthesis of family-centered care and partnership-in-care. J Pediatr Nurs.

[11] Nicholl H, Begley C (2012). Explicating caregiving by mothers of children with complex needs in Ireland: A phenomenological study. J Pediatr Nurs.

[12] Kuhlthau K, Kahn R, Hill KS, Gnanasekaran S, Ettner SL (2010). The well-being of parental caregivers of children with activity limitations. Matern Child Health J.

[13] Brenner M, Connolly M, Cawley D, Howlin F, Berry J, Quinn C (2016). Family and healthcare professionals’ perceptions of a pilot hospice at home programme for children: a qualitative study. BMC Palliative Care.

[14] Eaton N (2008). ‘I don’t know how we coped before’: A study of respite care for children in the home and hospice. J Clin Nurs.

[15] Remedios C, Willenberg L, Zordan R (2015). A pre-test and post-test study of the physical and psychological effects of out-of-home respite care on caregivers of children with life threatening conditions. Palliat Med.

[16] Dyches TT, Christensen R, Harper JM, Mandleco B, Roper SO (2016). Respite care for single mothers of children with autism spectrum disorders. J Autism Dev Disord.

[17] Harper A, Dyches TT, Harper J, Roper SO, South M (2013). Respite care, marital quality, and stress in parents of children with autism spectrum disorders. J Autism Dev Disord.

[18] Brenner M, Larkin P, Hilliard C (2015). Parents’ perspectives of the transition to home when a child has complex technological health care needs. Int J Integr Care.

[19] Deavenport-Saman A, Lu Y, Smith K, Yin L (2016). Do children with autism overutilize the emergency department? Examining visit urgency and subsequent hospital admissions. Matern Child Health J.

[20] Cohen-Silver JH, Muskat B, Ratnapalan S (2014). Autism in the emergency department. Clin Pediatr.

[21] Wilkie B, Barr O (2008). The experiences of parents of children with an intellectual disability who use respite care services. Learn Disabil Pract.

[22] Kalb LG, Stuart EA, Freedman B, Zablotsky B, Vasa R (2012). Psychiatric-related emergency department visits among children with an autism spectrum disorder. Pediatr Emerg Care.

[23] Soto EC, Frederickson AM, Trivedi H, Le A, Eugene MC, Shekher M, Weiskopf M, Allen-Dicker K, Dicker R, Fornari V, Correll CU (2009). Frequency and correlates of inappropriate pediatric psychiatric emergency room visits. J Clin Psychiatry.

[24] The Joanna Briggs Institute (2014). Joanna Briggs Institute Reviewers’ Manual: edition.

[25] Moher D, Liberati A, Tetzlaff J, Altman DG (2009). The PRISMA group. Preferred reporting items for systematic reviews and meta-analyses: the PRISMA statement. PLoS Med.

[26] Płatos M, Pisula E (2019). Service use, unmet needs, and barriers to services among adolescents and young adults with autism spectrum disorder in Poland. BMC Health Serv Res.

[27] Gray DE (2009). Doing research in the real world.

[28] Rahman MS (2017). The Advantages and Disadvantages of Using Qualitative and Quantitative Approaches and Methods in Language" Testing and Assessment" Research: A Literature Review. J Educ Learn.

[29] Kanner L (1943). Autistic disturbances of affective contact. Nervous Child.

[30] Brunton G, Wiggins M, Oakley A (2011). Becoming a mother: a research synthesis of women’s views on the experience of first-time motherhood.

[31] Mann AR (2013). The Experiences of Mothers of Children with Autism in Jamaica: An Exploratory Study of Their Journey. Graduate Theses and Dissertations.

[32] Hannes K, Lockwood C (2011). Pragmatism as the philosophical foundation for the Joanna Briggs meta-aggregative approach to qualitative evidence synthesis. 2011. J Adv Nurs.

[33] Keenan M, Dillenberger K, Doherty A, Byrne T, Gallagher S (2007). Meeting the needs of families living with children diagnosed with Autism Spectrum Disorder.

[34] Benderix Y, Nordström B, Sivberg B (2006). Parents' experience of having a child with autism and learning disabilities living in a group home: A case study. Autism..

[35] Minhas A, Vajaratkar V, Divan G, Hamdani SU, Leadbitter K, Taylor C, Aldred C, Tariq A, Tariq M, Cardoza P, Green J, Patel V, Rahman A (2015). Parents' perspectives on care of children with autistic spectrum disorder in South Asia - Views from Pakistan and India. Int Rev Psychiatry.

[36] Hodgetts S, Nicholas D, Zwaigenbaum L (2013). Home sweet home? families' experiences with aggression in children with autism spectrum disorders. Focus Autism Other Dev Disab.

[37] Gona JK, Newton CR, Rimba KK, Mapenzi R, Kihara M, Vijver FV, Abubakar A (2016). Challenges and coping strategies of parents of children with autism on the Kenyan coast. Rural Remote Health.

[38] Hartrey L, Wells JS (2003). The meaning of respite care to mothers of children with learning disabilities: two Irish case studies. J Psychiatr Ment Health Nurs.

[39] Mandell DS, Xie M, Morales KH, Lawer L, McCarthy M, Marcus SC (2012). The interplay of outpatient services and psychiatric hospitalization among Medicaid-enrolled children with autism spectrum disorders. Arch Pediatr Adolesc Med.

[40] Liu G, Pearl AM, Kong L, Douglas LL, Murray MJ (2017). A Profile on Emergency Department Utilization in Adolescents and Young Adults with Autism Spectrum Disorders. J Autism Dev Disord.

[41] Dew A, Buckley K, Veitch C, Bundy A, Lincoln M, Brentnall J, Gallego G, Griffiths S (2013). Carer and service providers’ experiences of individual funding models for children with a disability in rural and remote areas. Health Soc Care Commun.

[42] Cocks A (2000). Respite care for disabled children: Micro and macro reflections. Disabil Soc.

[43] McGill P, Papachristoforou E, Cooper V (2006). Support for family carers of children and young people with developmental disabilities and challenging behaviour. Child Care Health Dev.

[44] Pickard KE, Ingersoll BR (2016). Quality versus quantity: The role of socioeconomic status on parent-reported service knowledge, service use, unmet service needs, and barriers to service use. Autism..

[45] Health Service Executive National Review of Autism Services Past, Present and Way Forward. HSE. 2012a http://www.fedvol.ie/_fileupload/Next%20Steps/autismreview2012.pdf. Accessed on 17Jan 2018.

[46] Thomas KC, Parish SL, Rose RA (2012). Access to care for children with autism in the context of state Medicaid reimbursement. Matern Child Health J.

[47] Slainte Healthcare Report (2017). Department of Health. Dublin. https://www.gov.ie/en/campaigns/slaintecare-implementation-strategy/ Accessed 5 June 2018.

[48] Damiani G, Rosenbaum P, Swinton M, Russell D (2014). Frequency and determinants of formal respite care service use among caregivers of children with cerebral palsy in Ontario. Child Care Health Dev.

[49] MacDonald H, Callery P (2007). Parenting children requiring complex care: a journey through time. Child Care Health Dev.

[50] Mansell L, Wilson C (2009). Current perceptions of respite care: experiences of family and informal carers of people with a learning disability. J Intellect Disabil.

[51] Imran N, Chaudry MN, Azeem M, Bhatti MR, Choudhary ZL, Cheema MA (2011). A survey of Autism knowledge and attitudes among the healthcare professionals in Lahore, Pakistan. BMC Pediatr.

[52] Kogan MD, Blumberg SJ, Schieve LA, Boyle CA, Perrin JM, Ghandour RM (2009). Prevalence of parent-reported diagnosis of autism spectrum disorder among children in the US, 2007. Pediatrics.

[53] Golden S, Mason A (2008). and K. Spilsbury Systematic Searches for the Effectiveness of Respite Care. J Med Library Assoc.

